# Vasorelaxant and Antihypertensive Effects of Bergenin on Isolated Rat Aorta and High Salt-Induced Hypertensive Rats

**DOI:** 10.1155/2022/4886193

**Published:** 2022-11-22

**Authors:** Taseer Ahmad, Rahila Qayyum, Taous Khan, Mater H. Mahnashi, Mohammed M. Jalal, Malik A. Altayar, Osama M. Alshehri, Abdul Jabbar Shah

**Affiliations:** ^1^Department of Pharmacy, COMSATS University Islamabad, Abbottabad Campus, University Road, Abbottabad, KPK 22060, Pakistan; ^2^Laboratory of Cardiovascular Research and Integrative Pharmacology, College of Pharmacy, University of Sargodha, Sargodha 40100, Pakistan; ^3^Department of Pharmaceutical Chemistry, School of Pharmacy, Najran University, Najran, Saudi Arabia; ^4^Department of Medical Laboratory Technology, Faculty of Applied Medical Sciences, University of Tabuk, Tabuk, Saudi Arabia; ^5^Department of Clinical Laboratory Sciences, Collage of Applied Medical Science, Najran University, Najran, Saudi Arabia

## Abstract

Bergenin is a phenolic glycoside that has been reported to be present in some medicinal plants which are traditionally used for their antihypertensive actions. So, bergenin was investigated for antihypertensive and vasorelaxant experiments in a rat model. Bergenin produced a significant fall in the mean arterial pressure (MAP) of rats. To explore the involvement of NO and muscarinic receptors, rats were pretreated with L-NAME and atropine *in-vivo*. The L-NAME did not change significantly the effect of bergenin on MAP excluding the involvement of NO. Unlike the L-NAME, atropine pretreatment reduced the effect of bergenin on MAP, indicating the role of muscarinic receptors. In *in-vitro* study, the bergenin produced endothelium-dependent (at lower concentrations) and independent (at higher concentrations) vasorelaxation, which was attenuated significantly in the presence of atropine and indomethacin but not with L-NAME. While a partial response was observed against K^+^-induced contractions. This was further confirmed when bergenin partly shifted the CaCl_2_-CRCs toward right. Bergenin also suppressed the PE peak formation, indicating the antagonist effect against the release of Ca^2+^. Moreover, the bergenin-induced vasorelaxant response was not markedly attenuated with TEA, while significantly ablated with 4-AP and BaCl_2_. In conclusion, the antihypertensive effects of bergenin are due to Ca^2+^ channel blockade, K^+^ channels activation, and muscarinic receptor-linked vasodilation.

## 1. Introduction

Medicinal plants and their phytochemical constituents have been documented as potential sources of therapeutic agents [1]. It has been reported that 30%–50% of all marketed drugs have their origin from medicinal plants [2]. Major classes of phytochemicals are reported for different pharmacological activities including, glycosides, alkaloids, and polyphenols [3].

**Figure 1:**
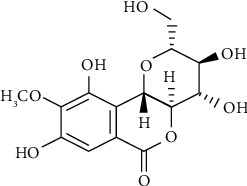
The chemical structure of bergenin.

Bergenin is a c-glucoside of 4-O-methylgallic acid/trihydroxybenzoic acid glycoside (Figure 1) [4]. Bergenin is a phenolic glycosides due to gallic acid (a phenolic compound) in its structure. It reveals a wide range of pharmacological activities and also in numerous cases is responsible for the folk use of its natural sources [5]. Bergenin has been reported to occur as a major constituent in several *Bergenia* species like *Bergenia crassifolia*, *Bergenia stracheyi*, and *Bergenia ligulata* Wall, which are reported and traditionally used for their antioxidant and antihypertensive effects [6–9]. Another major source of bergenin is *Ficus racemosa L*, which is reported for its antioxidant and angiotensin-converting enzyme inhibitory effect [4]. However, earlier reported activities have not recognized the active constituents responsible for antihypertensive activity and could not reach to the decisive mechanism.

Bergenin is reported for several biological activities, including antiulcer [10], antiplatelet [11], antioxidant [12], antiarthritic [13], anti-inflammatory activity [13, 14], and hypolipidemic activities [15]. However, bergenin is not investigated as an antihypertensive agent. This study was intended to identify the role of bergenin against hypertension and its probable vascular mechanisms.

## 2. Materials and Methods

### 2.1. Chemicals and Reagents

The reference chemicals, acetylcholine chloride, angiotensin II (Ang II), atropine sulfate, BaCl_2_, dimethyl sulfoxide (DMSO), phenylephrine hydrochloride, potassium chloride, indomethacin, N*ω*-Nitro-L-arginine methyl ester (L-NAME), tetraethylammonium chloride (TEA), 4-aminopyridine (4-AP), verapamil hydrochloride and test compound bergenin, EGTA, thiopental sodium, and heparin inj. were purchased from specified standard resources. For most drugs, distilled water/normal saline is used as a solvent; however, ethanol is used as a solvent for indomethacin and bergenin was first dissolved in DMSO and then diluted with distilled water (the final bath concentration for *in-vitro* study was <0.1% DMSO and *in-vivo* study doses contain ≤1% DMSO).

### 2.2. Experimental Animals

Antihypertensive and vascular reactivity study was conducted on adult male Sprague-Dawley (SD) rats of weight 200–250 g that were placed under the standard conditions of the animal house of CUI, Abbottabad campus, Abbottabad (60% humidity, 23 ± 1°C) with a 12 h dark/light schedule. The ethical committee of the Pharmacy department (CUI, Abbottabad campus, Abbottabad) approved this protocol in a meeting held on June 18, 2013 (notification # EC/PHM/07–2013/CUI/ATD).

### 2.3. Measurement of Invasive Blood Pressure

#### 2.3.1. Measurement of MAP in Normotensive SD Rats

These experiments were carried out according to the protocol followed by Shah and Gilani, (2009) [16] and Taqvi et al. (2008) [17] with few changes. SD rats were anaesthetized with administration of pentothal (≈60 mg/kg i.p). After that, approximately, 1 cm mid-tracheal incision was made and trachea was cannulated with PE-20, while PE-50 was inserted in the left carotid artery and right jugular vein. This cannulation was important for BP recording. To record and analyze the BP, invasive BP apparatus (ADInstruments) was used. When the animal is stable (after 20–30 min), the hypertensive and hypotensive responses of animal were checked by norepinephrine and acetylcholine (1 *µ*g/kg of each). After that different doses of bergenin were injected. Standard experimental drugs like L-NAME (20 mg/kg) and atropine (1 mg/kg) were used to identify the role of nitric oxide (NO) pathway and muscarinic receptors. Then MAP was calculated according to the standard formula [18].

#### 2.3.2. Effect of Bergenin on MAP of the High Salt (8%) Hypertensive Rat Model

A high salt diet (8% NaCl in water and food for 14 days) was used to induce hypertension in normotensive rats. Rats were considered hypertensive with systolic BP > 140 mmHg and diastolic BP more than 90 mmHg. The rest protocol was same as mentioned for normotensive rats [18, 19].

### 2.4. Vascular Reactivity Studies

#### 2.4.1. Tension Studies in Isolated Rat Aorta

The isolated SD rat aorta was to see the vascular reactivity response of bergenin. The 2 mm aortic ring after cleaning from extra tissues was transferred to the 10 mL bath, aerated with carbogen, and the temperature was maintained at 37°C. A tension of 2 g was applied after hanging tissue in the bath. The stability period was almost 45 min. During this period, the tissue was washed after every 15 min. The response in aortic ring was recorded through PowerLab attached with an amplifier and transducer (ADInstruments) [19].

#### 2.4.2. Determination of Bergenin Response in the Presence of Different Vessel-Related Signaling Pathway Inhibitors

Initially, the vasorelaxant response of bergenin was confirmed against the phenylephrine (1 *μ*M) induced contraction in endothelium intact aortic tissues. To differentiate the role of endothelium, some tissues were deliberately denuded. Furthermore, standard experimental drugs, L-NAME (10 *µ*M), atropine (1 *µ*M), and indomethacin (1 *µ*M), were added to intact rat aortic rings to determine the involvement of nitric oxide (NO), muscarinic receptor, and prostacyclin in the relaxation response. The mentioned experimental drugs were added 20 min prior to the addition of phenylephrine. Responses were compared in the presence and absence of the abovementioned inhibitors [18, 20].

#### 2.4.3. Effect of Bergenin on Ca^2+^ Signaling Pathways

The procedures suggested by Furchgott and Zawadzki [21] and Ahmad et al. [18] were adopted with some changes. Phenylephrine (1 *µ*M), K^+^ (80 mM), and Ang II (5 *µ*M) in separate experiments were added to the rat aortic rings for obtaining steady-state contractions. After that, bergenin was added at different concentrations cumulatively and the response was observed (in a separate set of experiments). To observe the effect of bergenin on calcium channels, concentration response curves (CRCs) of CaCl_2_ (0.01–10.0 mM) (as Ca^2+^) were produced in the presence of bergenin in a calcium-free medium. In addition, the effect of bergenin on intracellular calcium stores was also confirmed by producing phenylephrine individual contraction in calcium-free Kreb's solution.

#### 2.4.4. The Effect of Bergenin on K^+^ Channels

Contractile responses were obtained by adding phenylephrine in both the absence (control) and presence of potassium channel blockers; tetraethylammonium (TEA) (5 mM) [22], 4-aminopyridine (4-AP) (1 mM) [23], and barium chloride (BaCl_2_) (30 *µ*M) [24] in different experiments, 20 min prior to phenylephrine-induced contraction. The response of bergenin was obtained by adding different concentrations cumulatively.

### 2.5. Statistical Analysis

GraphPad Prism (8) was used for statistical analysis. Student's *t*-test and two-way ANOVA (Bonferroni test) were applied for data analysis. The data were reflected as significant when ^*∗*^*p* ≤ 0.05.

## 3. Results

### 3.1. Antihypertensive Activities of Bergenin

#### 3.1.1. Blood Pressure Lowering Effect of Bergenin in Both Normotensive and Hypertensive Rats

**Figure 2:**
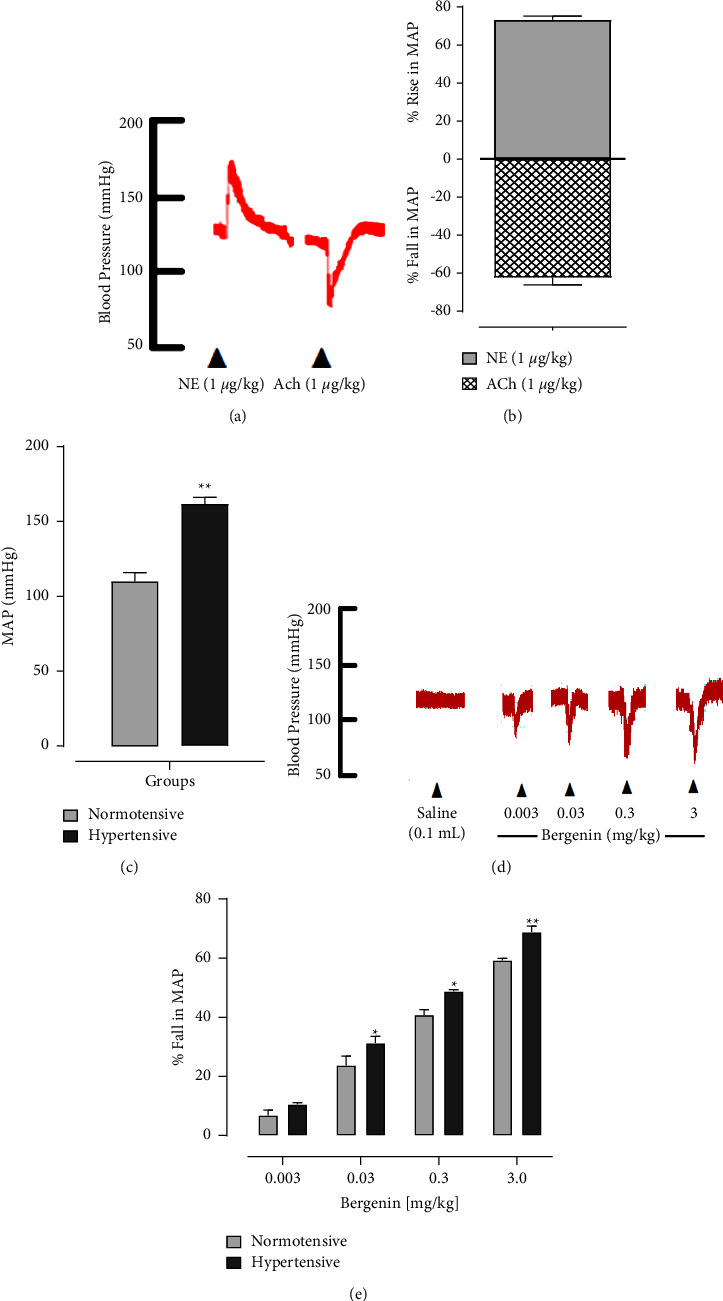
A representative tracing. (a) shows the response of norepinephrine (NE) and acetylcholine (ACH) on MAP and (b) reveals the % increase and fall in BP of normotensive rats. (c) shows the response of NE and ACH on MAP in both normotensive and hypertensive rats. (d) A representative tracing showing the effect of bergenin on BP in normotensive anaesthetized rats. The bar graph (e) shows the fall in MAP produced by bergenin in normotensive and hypertensive rats. ^*∗*^*p* < 0.05 and ^*∗∗*^*p* < 0.01 describe the significant differences.

Intravenous (i.v) injections of norepinephrine (1 *µ*g/kg) and acetylcholine (1 *µ*g/kg) produced a significant increase and decrease in the MAP of both anaesthetized normotensive and hypertensive SD rats, respectively (Figures 2(a)–2(c)). The MAP calculated for the normotensive and hypertensive rats was 115 ± 2.09 mmHg and 163 ± 2.18 mmHg (Figure 2(c)). These measures validated the protocols. Bergenin produced a graded dose-response by decreasing the MAP both in normotensive and hypertensive rats, respectively (Figure 2(e)). The % decrease in MAP was 6.01 ± 0.44, 23.75 ± 1.33, 40.75 ± 1.30, and 59.25 ± 2.10 mmHg at 0.003^_^3 mg/kg doses, as shown in Figures 2(d) and 2(e). Bergenin produced a more significant fall in MAP of hypertensive rats that was 10.50 ± 0.9, 31.50 ± 1.45, 48.75 ± 2.84, and 68.75 ± 2.52 mmHg, as shown in Figure 2(e). In the normotensive and hypertensive rats treated with different doses of bergenin induced a significant decrease in the heart rate (48%, 56% at 3 mg/kg dose) associated with a fall in blood pressure, as shown in Table 1.

#### 3.1.2. Effects of Bergenin on MAP in SD Rats in the Presence of L-NAME and Atropine

**Figure 3:**
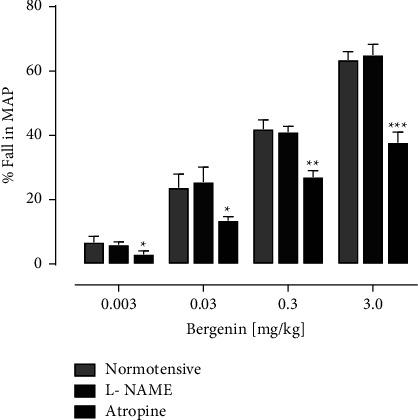
Comparison of % decrease in MAP by bergenin in normotensive, pretreated L-NAME (20 mg/kg) and atropine (1 mg/kg) normotensive SD rats. While ^*∗*^*p* < 0.05, ^*∗∗*^*p* < 0.01 and ^*∗∗∗*^*p* < 0.001, exhibits the significance. mean ± SEM (*n* = 6).

The experiments were carried out in anaesthetized normotensive SD rats. Before the injection of bergenin, L-NAME (20 mg/kg) and atropine (1 mg/kg) were preadministered. The L-NAME pretreatment did not significantly alter changes in the MAP to bergenin; 6.0 ± 0.95, 25.50 ± 0.80, 41.0 ± 2.80, and 65.0 ± 3.27 mmHg (Figure 3). While in the atropine pretreated rats, the magnitude of the fall in the MAP to bergenin was reduced as 3.01 ± 0.90, 17.50 ± 1.81, 27.0 ± 2.30, and 39.50 ± 3.60 mmHg (Figure 3).

### 3.2. Stud on Isolated Blood Vessels

#### 3.2.1. Effect of Bergenin on Isolated Rat Aortic Tissues

**Figure 4:**
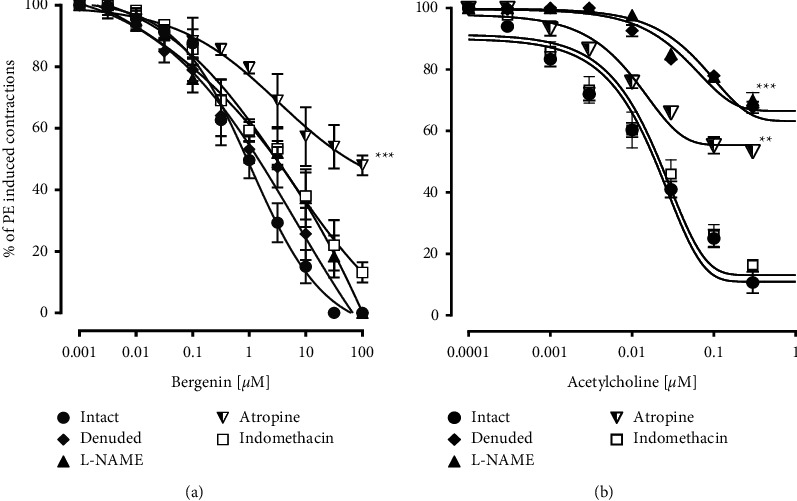
Effect of (a) bergenin and (b) acetylcholine on phenylephrine (PE; 1 *µ*M) pre-contractions in intact, denuded, pretreated; 10 *µ*M L-NAME, 1 *µ*M atropine, and 1 *µ*M indomethacin on rat aortic rings. The relaxation responses, shown as means ± SEM (*n* = 6) where ^*∗∗*^*p* < 0.01 and ^*∗∗∗*^*p* < 0.001, represent the significance difference.

The contraction was induced in intact aortic rings by preincubation with phenylephrine (1 *μ*M), followed by the cumulative addition of bergenin. This resulted in a vasorelaxant response with an EC_50_ value of 1.09 *μ*M (0.90–2.06) (Figure 4(a)). Moreover, in denuded tissues, the response of bergenin was not changed significantly (at higher concentrations), with EC_50_ values 1.70 *μ*M (1.95–2.65) (Figure 4(a)). This confirms the nonsignificant role of factors related to endothelium. This response is further validated by the unchanged vasorelaxant response of bergenin against the phenylephrine-induced contractions in isolated tissues, preincubated with 10 *µ*M L-NAME. The EC_50_ value was 1.85 *μ*M (1.98–3.01) (Figure 4(a)). The pretreatment of atropine significantly inhibited the vasorelaxant effect of bergenin (>50%) (Figure 4(a)). Moreover, the indomethacin pretreatment partially modifies the effect of bergenin with the EC_50_ value, 3.35 *μ*M (1.60–4.41) (Figure 4(a)). The effect of bergenin is compared with acetylcholine (Figure 4(b)).

**Figure 5:**
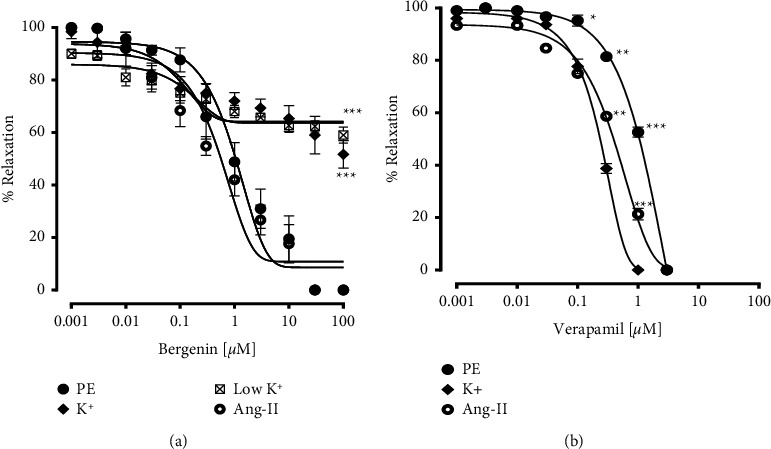
Vasorelaxant response of (a) bergenin and (b) verapamil on phenylephrine (PE; 1 *µ*M), high K+ (80 mM), low K+ (20 mM) and 5 *µ*M Ang II precontractions. The relaxation responses are shown as means ± SEM (*n* = 6), where ^*∗*^*p* < 0.05, ^*∗∗*^*p* < 0.01 and ^*∗∗∗*^*p* < 0.001 vs. control group.

Moreover, bergenin induced concentration-dependent relaxation in comparison to verapamil against the contraction induced by phenylephrine, and Ang II in isolated tissues with EC_50_ values of 1.14 *μ*M (0.90–1.87) and 0.63* μ*M (0.50–1.20), respectively. However, the bergenin vasorelaxant response was highly reduced against the pre-contractions induced by both 80 mM (49%) and 20 mM KCl (39%), as shown in (Figures 5(a) and 5(b)).

#### 3.2.2. Calcium Channels' Antagonist Effect of Bergenin

**Figure 6:**
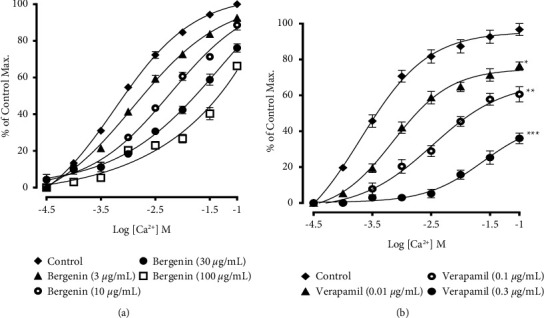
Calcium antagonist response of (a) bergenin and (b) verapamil on the CRCs (concentration response curves) produced in Ca^2+^-free/EGTA solution. Contractile responses, shown as means ± SEM (*n* = 6), where ^*∗∗*^*p* < 0.01 and ^*∗∗∗*^*p* < 0.001 vs. control group.

In calcium-free medium, the cumulative addition of different concentrations (3–100 *μ*M) of bergenin significantly shifted the concentration response curves (CRCs), induced by calcium chloride (CaCl_2_), toward the right (Figure 6(a)). This response of bergenin was compared to verapamil (0.01─ 0.3 *μ*M) (Figure 6(b)).

#### 3.2.3. Bergenin Attenuated the Intracellular Calcium Stores

**Figure 7:**
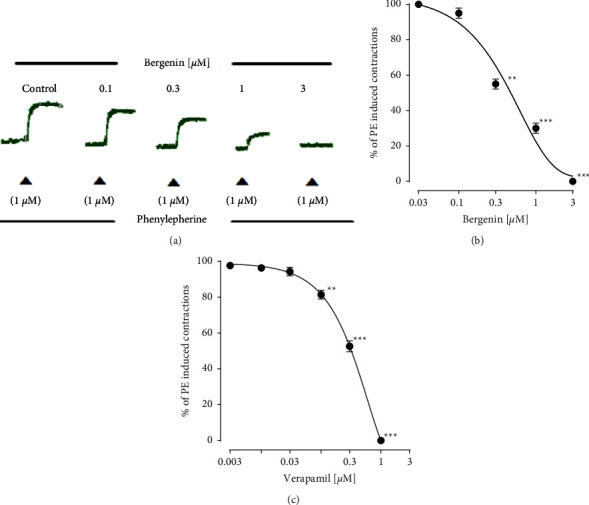
A representative tracing (a) shows inhibitory responses of different concentrations of bergenin against the phenylephrine peaks in calcium-free solution. The graphs show the increasing concentrations of (b) bergenin and (c) verapamil and their effect on the individual contraction of phenylephrine in a calcium-free medium. The relaxation responses are shown as means ± SEM (*n* = 6), where ^*∗∗*^*p* < 0.01 and ^*∗∗∗*^*p* < 0.001.

Pre-incubation of bergenin (0.1–3.0 *µ*M) produced a significant inhibitory response against the intracellular calcium, by suppressing the individual contractions produced by phenylephrine in calcium-free medium. This response of bergenin was compared to verapamil (Figures 7(a)–7(c)).

#### 3.2.4. Bergenin Response in the Presence of Potassium Channel Inhibitors

**Figure 8:**
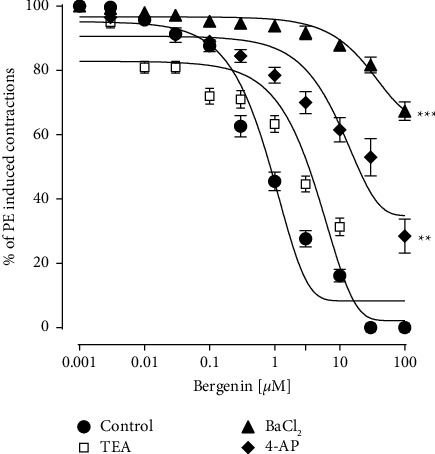
The response of bergenin on phenylephrine (1 *µ*M) pre-contractions in control and pretreated; tetraethylammonium (TEA; 5 mM), 4-aminopyridine (4-AP; 1 mM) barium chloride (BaCl_2_; 30 *µ*M) rat aortic rings. The relaxation responses are shown as means ± SEM (*n* = 6). Where ^*∗∗*^*p* < 0.01 and ^*∗∗∗*^*p* < 0.001 vs. control group. vs. control group.

To identify the role of potassium channels in the response produced by bergenin, different potassium channel inhibitors; TEA, BaCl_2_, and 4-AP were used. In the presence of TEA (5 mM), the vasorelaxant response of bergenin was not changed significantly. However, 4-AP and BaCl_2_ significantly (23%, 69%) attenuated the bergenin response (Figure 8).

## 4. Discussion

In this study, the response of bergenin against blood pressure was investigated both in normotensive and hypertensive rats. In addition to the *in-vivo* measurement of MAP in normotensive rats, BP measurement in hypertensive rats is considered the most authentic approach. Due to this reason, bergenin is also evaluated in the hypertensive model. In the 8% salt hypertensive model, bergenin produced a significant decrease in MAP. However, the % fall in MAP in hypertensive rats was higher as compared to normotensive rats. This might support the hypothesis that drugs produced a more potent response in pathological conditions. After these exciting findings on bergenin, as an antihypertensive agent, further mechanistic studies were carried out. In denuded tissues, the bergenin response was not completely blocked, although less potent relaxation was observed as compared to control (intact aortic tissues). To comprehend the nitric oxide (NO)-pathway involvement in the in the antihypertensive response of bergenin, the L-NAME was preinjected in SD rats, however, no significant change in the blood pressure lowering response of bergenin was observed. The other possibility was that, bergenin might produce its effect through muscarinic receptors. So, to confirm the role of muscarinic receptors, we used atropine to inhibit the muscarinic receptors [25, 26]. This pre-administration modifies (26%) the effect of bergenin on MAP, which shows that bergenin has an inhibitory effect on vascular muscarinic receptors. These results confirmed that bergenin is one main agent present in its plant sources which are reported for their antihypertensive effects, like *Bergenia crassifolia* leaves' extract is reported for its hypotensive effect in rats and *Bergenia ligulata* Wall in dogs. Moreover, bergenin produced a significant fall (50%) in the heart rate (HR), which might be due to the Ca^2+^ antagonist activity. This response of bergenin is also comparable to verapamil. So, further studies are suggested to trace this negative chronotropic effect in a perfused isolated rat heart model. Interestingly, the bergenin plant source, the *Bergenia ligulata* Wall extract is also reported for negative inotropic and chronotropic effects [6, 8, 27]. To further study the response of bergenin on vascular mechanism (s) linked to hypertension, isolated rat aorta was used for further *in-vitro* studies.

Initially, some standard vasoconstrictors were used like phenylephrine, high K^+^, and Ang II, respectively. The contraction produced by phenylephrine and Ang II was significantly reduced (100%) by bergenin, while a partial response was observed against the high K^+^ (49%) and even at low K^+^ (20 mM; 39%) contractions. This response confirms initially the calcium antagonist effect of bergenin.

To investigate the endothelium-dependent and independent response different experiments were performed. The relaxation to bergenin was partially reduced (at initial concentration), while at higher concentrations, no significant change in the response was observed in aortic rings with pretreatment of L-NAME, a nitric oxide inhibitor [28]. These findings excluded the dominant role of nitric oxide (NO). In vascular endothelial muscarinic receptors (*M*_3_) also have a role in vasorelaxation, to observe its involvement in the response produced by bergenin, atropine was preincubated [26]. This preincubation of atropine reduced (54%) the vasorelaxant effect of bergenin. So, muscarinic receptors are partially involved in the vasorelaxant effect of bergenin. Other endothelium-linked vasoactive substances include a prostacyclin inhibitor, indomethacin [29, 30]. With preincubation of indomethacin, a partial change in the vasorelaxant (18%) response of bergenin was observed.

As confirmed before initially that bergenin produced a vasorelaxant response against the contraction produced by phenylephrine, suggesting a Ca^2+^ inhibitory response against the intracellular Ca^2+^. Phenylephrine is well known for its biphasic contraction. A sharp contraction (fast phase) followed by a stable contraction (slow phase), due to Ca^2+^ release from the stores and then influx of Ca^2+^ through receptors operated calcium channels (ROCCs) [31]. This response was further validated by the inhibitory effect of different concentrations of bergenin against the phenylephrine individual peaks. Such a response was also observed with selected standard Ca^2+^ entry blocker verapamil [21].

In aggregate, the vasorelaxant response of bergenin is mediated through its inhibitory action on the IP_3_-dependent Ca^2+^ pathway which is sensitive to phenylephrine contraction. These findings encouraged us to investigate the response of bergenin against the voltage gated Ca^2+^ channels present in the plasma membrane. As discussed previously that bergenin produced a partial response against contraction induced by high K^+^. Moreover, the contraction is induced by high K^+^ through the opening of L-type calcium channels [31, 32]. So, drugs that inhibit high K^+^ precontraction can be considered as a calcium channel antagonist [33]. A partial vasorelaxant response was observed with bergenin against the 20 and 80 Mm K^+^ precontractions on isolated rat aorta, in comparison to verapamil. To investigate further, rat aortic rings were hung in a calcium-free solution. Then, preincubation of the isolated tissues with different concentrations of bergenin induced a partial rightward shift in CRCs produced by CaCl_2_ addition, in comparison to verapamil, indicating that bergenin inhibits partly the Ca^2+^ entry through VDCs. The response of bergenin was further investigated.

Previous studies have confirmed that Ang II receptors are present in rat aortic smooth muscle cells and play a vital role in marinating the tone of blood vessels [34, 35]. So, bergenin was added cumulatively against the precontraction produced by Ang II in rat aortic tissues. In response, a significant vasorelaxant response was observed, which suggests further studies to identify the exact target of bergenin in the Ang II-produced signaling pathway.

To have further insights into the response produced by bergenin, the role of potassium channels was also investigated. Potassium channels in the vascular smooth muscles play a vital role in vascular activity and blood pressure. Different types of potassium channels included; Ca^2+^-activated K^+^ channels (K_Ca_), inward rectifying K^+^ channels (Kir), and K^+^ voltage-gated channels (Kv), respectively. The pretreatment of BaCl_2_ (Kir channels inhibitor) [36] and 4-AP (Kv channels inhibitor) [37] significantly (69% and 23%) reduced the vasorelaxant effect of bergenin. The TEA, blocker of K_Ca_ channels [38], was unable to block significantly the effect of bergenin. In aggregate, the involvement of potassium channels (Kv and Kir) can be considered in the predominant endothelium-independent vasorelaxant response of bergenin.

## 5. Conclusion

So, these findings have identified glycoside bergenin as a potential antihypertensive agent. Our data revealed that bergenin exerts its hypotensive effect through its vasodilatory potential. Findings on the antihypertensive and vascular reactivity response of bergenin are mainly mediated through its action on muscarinic receptors, attenuation of Ca^2+^ intracellular stores and opening of potassium channels which possibly explain the underlying mechanisms.

## Figures and Tables

**Table 1:** Reveals the percent decrease in the BP and heart rate (HR) with different doses of bergenin in rats.

Dose (mg/kg)	*Normotensive rats*		*Hypertensive rats*	
	BP (%)	HR (%)	BP (%)	HR (%)
Control	99.9 ± 0.06	99.4 ± 0.04	99.2 ± 0.10	99.7 ± 0.07
0.003	7 ± 0.64^*∗*^	20 ± 1.23^*∗*^	10.50 ± 0.93	25 ± 1.02^*∗*^
0.03	24 ± 2.28^*∗*^	25 ± 1.84^*∗*^	31.50 ± 1.45	28 ± 3.04^*∗*^
0.3	42 ± 0.62^*∗∗*^	40 ± 1.03^*∗∗*^	48.75 ± 2.84	39 ± 2.04^*∗∗*^
3	58 ± 2.05^*∗∗∗*^	48 ± 2.30^*∗∗∗*^	68.75 ± 2.52	56 ± 3.14^*∗∗∗*^

Values were tabulated as mean ± SEM for six experiments, where ^*∗*^*p* < 0.05, ^*∗∗*^*p* < 0.01 and ^*∗∗∗*^*p* < 0.001 vs. Control.

## Data Availability

The data used to support the findings of this study are available on request from the corresponding author Dr. Abdul Jabbar Shah; e-mail: jabbarshah@cuiatd.edu.pk.
